# Fatty acid composition and anticancer activity in colon carcinoma cell lines of *Prunus dulcis* seed oil

**DOI:** 10.1080/13880209.2017.1296003

**Published:** 2017-03-05

**Authors:** Filiz Mericli, Eda Becer, Hilal Kabadayı, Azmi Hanoglu, Duygu Yigit Hanoglu, Dudu Ozkum Yavuz, Temel Ozek, Seda Vatansever

**Affiliations:** a Department of Pharmacognosy-Phytotherapy, Faculty of Pharmacy, Near East University, Mersin, Turkey;; b Department of Biochemistry, Faculty of Pharmacy, Near East University, Mersin, Turkey;; c Department of Histology and Embryology, Faculty of Medicine, Celal Bayar University, Manisa, Turkey;; d Department of Pharmaceutical Botany, Faculty of Pharmacy, Near East University, Mersin, Turkey;; e Department of Pharmacognosy, Faculty of Pharmacy, Anadolu University, Eskisehir, Turkey;; f Experimental Health Research Center of Health Sciences, Near East University, Mersin, Turkey

**Keywords:** Almond oil, oleic acid, colo-320 cell line, colo-741 cell line

## Abstract

**Context:** Almond oil is used in traditional and complementary therapies for its numerous health benefits due to high unsaturated fatty acids content.

**Objectives:** This study investigated the composition and *in vitro* anticancer activity of almond oil from Northern Cyprus and compared with almond oil from Turkey.

**Materials and methods:** Almond oil from Northern Cyprus was obtained by supercritical CO_2_ extraction and analyzed by GC-MS. Almond oil of Turkey was provided from Turkish pharmacies. Different concentrations of almond oils were incubated for 24 and 48 h with Colo-320 and Colo-741 cells. Cell growth and cytotoxicity were measured by MTT assays. Anticancer and antiprolifetarive activities of almond oils were investigated by immunocytochemistry using antibodies directed against to BMP-2, β-catenin, Ki-67, LGR-5 and Jagged 1.

**Results:** Oleic acid (77.8%; 75.3%), linoleic acid (13.5%; 15.8%), palmitic acid (7.4%; 6.3%), were determined as the major compounds of almond oil from Northern Cyprus and Turkey, respectively. In the MTT assay, both almond oils were found to be active against Colo-320 and Colo-741 cells with 1:1 dilution for both 24 h and 48 h. As a result of immunohistochemical staining, while both almond oils exhibited significant antiproliferative and anticancer activity, these activities were more similar in Colo-320 cells which were treated with Northern Cyprus almond oil.

**Discussion and conclusion:** Almond oil from Northern Cyprus and Turkey may have anticancer and antiproliferative effects on colon cancer cells through molecular signalling pathways and, thus, they could be potential novel therapeutic agents.

## Introduction

The almond *Prunus dulcis* (Mill) D.A. Webb (subfamily Prunoideae of family Rosaceae) is native to Mediterranean countries and other hot climates regions (Davis 1970; Askın et al. 2007; Kodad et al. 2014). Almond seeds contain fixed oil, phenolic compounds, and some micronutrients, vitamins, minerals and have different biological activities (Ying et al. 2015). Almond seeds and oil have anti-inflammatory, immunostimulant effects, and reduce irritable bowel syndrome symptoms, and they are also useful for treating constipation (Lardos 2006; Esfahlan et al. 2010; Zeeshan 2010). Almond oil has been also used to treat dry skin disorders such as psoriasis and eczema in ancient treatment cultures. Today it is used in aromatherapy massage applications as carrier oil, and also producing many skin-hair cosmetics (Buckle 2002). Almond oil is rich in unsaturated fatty acids, mainly oleic acid and linoleic acid and, when added to the diet, increase HDL-cholesterol and reduce LDL-cholesterol, improve body weight control, and reduce the risk of obesity-related health disorders such as heart disease and type II diabetes (Hollis & Mattes 2007; Damesceno et al. 2011). Hepatoprotective and anticancer activity of almond oil was reported by some *in vivo* research (Davis & Iwahashi 2001; Jia et al. 2011).

The importance of the fatty acids constituents of nuts in the maintenance of health and protection from cancer is also of interest. Especially, animal studies have provided evidence that almond oil is closely associated with reduction in the incidence of colon cancer (Davis & Iwahashi 2001). The specific effects of almond oil with respect to signalling molecules which play a role during tumour viability metastasis, transcription factors and cell proliferation in colon carcinoma cells remain undefined. There are no reports addressing the effect of almond oil on colon cancer with *in vitro* and *in vivo* studies.

In Cyprus, almond trees are widespread and unripe-soft and green fruits are eaten and Cypriot people prepare some special sweet food named ‘*somata*’ using almond seeds. The oil is used for skin care and treating skin diseases (abscess, furuncle, scabies, skin ulcer, etc.) and also tenesmus and constipation (Lardos 2006).

This study determines the fatty acid composition and *in vitro* anticancer activity of almond oil from Northern Cyprus (NC) compared to almond oil from Turkey (TR) in primary (Colo-320) and metastatic (Colo-741) colon carcinoma cells. In addition, we aimed to examine almond oil-mediated antiproliferative effects which decrease of Ki-67 expression and its protective effects toward relevant molecular mechanisms, including BMP-2, β-catenin, Jagged 1 and LGR-5 that plays a role during tumour viability metastasis and transcription factors.

## Materials and methods

### Almond seeds and almond oil

Almond seeds were obtained from almond trees of Yedidalga village, Northern Cyprus in August 2015. Almond oil was obtained by using supercritical CO_2_ extraction (Super critical fluid extraction 100-2- FMC system) (Thar Instruments Inc.) (Marrone et al. 1998). Turkish origin almond oil was obtained from Turkish Pharmacies (Almond Oil, DD. BY05 expiration date: 4/4/2018).

### GC-MS analysis of almond oil

Fatty acid composition of almond oil samples were investigated by GC-MS analysis. Almond oil was methylated according to the method reported previously (Goren et al. 2006). The fatty acid methyl esters were analyzed using an Agilent 5975C VL MSD with a triple-axis detector system and an Agilent 7890A GC system. The column was HP-Innowax FSC (60 m × 0.25 mm, 0.25 μm film thickness). Carrier gas was helium (1.4 mL/min). GC oven temperature was programed as 10 min at 60 °C/4 °C/min to 220 °C/10 min at 220 °C/1 °C/min to 240 °C/20 min at 240 °C (total 100 min). One microliter sample (10% in hexane v/V) was injected into the system. Injection mode: split mode (40:1). Injector temperature: 250 °C. Transfer line temperature: 280 °C. Mass spectrum: 70 eV. Mass range: *m/z* 35 to 450 GC Detector: FID at 300 °C. In order to obtain the same elution order with GC-MS, the column outlet was split into two, one for FID and the other for MS detector.

### Identification and quantification of compounds

Identification of the volatile constituents was achieved by parallel comparison of their retention indices and mass spectra with data stored in the Wiley/NIST GC/MS Library (W908N.L, New York, NY). *n*-Alkanes (C8-C40) were used as reference points in the calculation of retention indices (RI). Quantification of volatiles components was performed on the basis of their GC/FID peak areas using integration data.

### Cell line and cell culture

Colo-320 (human colon adenocarcinoma, ATCC catalog: CCL 220) and Colo-741 cell lines (ECACC 93052621) were maintained in culture in RPMI-1640 medium (Biochrom, FG1215), 10% FBS (Capricorn Scientific, FBS-11B), 1% penicillin-streptomycin (Biochrom, A2213) and 1% glutamine (EMD Millipore, K0282). Cells were cultured in a humidified atmosphere at 37 °C in 5% CO_2_. When the cells were 80% confluent, they were routinely subcultured using 0.25% trypsin-EDTA solution (Biochrom, L2143).

### Cell viability and growth assays

The MTT assay, reduction of 3-(4,5-dimethylthiazol-2-yl)-2,5-diphenyltetrazolium bromide to a purple formazan product, was used to estimate cell viability and growth. Pure almond oil diluted in culture medium with four dilutions (1:1, 1:2, 1:4, 1:8, 1:16). Cell suspensions were first prepared at densities of 5 × 104/mL cells per each well of 96-well culture dishes and plated in triplicate for each oil concentration. Medium (100 μL) without almond oil was used as a positive control, and only medium which did not contain any cells and almond oil was used as a negative control.

Colo320 and Colo741cells were treated with the concentrations previously mentioned for 24 and 48 h. Cells were incubated in humidified 5% CO_2_ (in air) at 37 °C with MTT in the last 2 h of the culture period tested. The medium was then decanted and 200 μL dimethylsulfoxide (DMSO, Sigma-Aldrich) was added to each well to ensure dissolving of the formazan salts. The absorbance was immediately determined at 570 nm in an UV–visible spectrophotometer multiplate reader (Versa Max, Molecular Device, Sunnyvale, CA).

### Cultivation of cells with pure almond oil from Northern Cyprus and Turkey

Cells were divided into three groups; the first group was the control group which was cultured with standard culture medium. The second group was treated with 1:1 of almond oil from NC, and third group was treated with 1:1 almond oil from TR according to MTT assay analyses result for 24 or 48 h.

### Immunocytochemistry

Cultures were also assessed immunocytochemically for binding of antibodies against bone morphogenetic proteins 2 (BMP-2), β-catenin, leucine-rich repeat containing G protein-coupled receptor 5 (LGR-5), jagged 1, Ki-67. Cells from all the groups were fixed with 4% paraformaldehyde in phosphate buffered saline (PBS) at 4 °C for 30 min. Next, Tween 20 (Sigma Aldrich) was added for 15 min for permeabilization. After washing with PBS, endogenous peroxidase activity was quenched by incubation with 3% H_2_O_2_ for 5 min at room temperature. Cells were then washed with PBS, and incubated with primary antibodies: anti-BMP-2 (orb251474, Biorbyt), anti-β-catenin (sc-59737, Santa Cruz Biotechnology Inc.), anti-LGR5 (HPA012530, Atlas Antibodies), anti-jagged 1 (sc-8303, Santa Cruz Biotechnology Inc.) and anti-Ki-67 (sc-15402, Santa Cruz Biotechnology Inc.) all overnight at 40 °C. The cells were then incubated with secondary antibodies according to manufacturer protocols (Histostain-Plus IHC Kit, HRP, 859043, ThermoFisher). They were then incubated with diaminobenzidine (DAB) for 5 min to visualize immuno-labelling. They were washed with PBS and counterstained with Mayer’s hematoxylin for 1 min and mounted with mounting medium (Merck Millipore-107961, Germany). All specimens were then evaluated under a light microscope (Olympus BX40, Tokyo, Japan).

Staining of BMP-2, β-catenin, LGR-5, jagged 1 and Ki-67 was also graded semiquantitatively using the H-SCORE that was calculated with the following equation: HSCORE = Σ*л* (*i* + 1), where *i* is the intensity of staining with a value of 1, 2 or 3 (mild, moderate, or strong, respectively) and *л* is the percentage of epithelial cells stained with each intensity, varying between 0 and 100%.

### Analysis of the data

Results were expressed as mean ± standard deviation (SD). Differences among groups were analyzed statistically with one-way ANOVA where appropriate. A *p*-value of <0.05 was considered significant.

## Results

### Analysis of almond oils

Almond seeds from NC yielded 38.8% fixed oil. Major compounds of that oil were determined as oleic acid (77.8%), linoleic acid (13.5%) and palmitic acid (7.4%). Almond oil from TR (DD.BY05) contains oleic acid (75.3%), linoleic acid (15.8%), palmitic acid (6.3%) and palmitoleic acid (0.6%). The fatty acid composition of the almond oil samples is given in Table 1.

**Table 1.** Fatty acid composition of almond oils from Northern Cyprus (NC) and Turkey (TR).

			Percentage	
	C number: double bond number	Lipid name	NC	TR
Palmitic acid (=Hexadecanoic acid)	C16:0 D		7.4	6.2
Palmitoleic acid (= (*Z*)-9-Hexadecenoic acid	C16:1 D 9 cis	Omega-7	0.4	0.6
Oleic acid (= (*Z*)-9-Octadecenoic acid)	C18:1 D 9 cis	Omega-9	77.8	75.3
Elaidic acid (= (*E*)-9-Octadecenoic acid)	C18:1 D 9 trans	Omega-9	0.3	0.1
Linoleic acid (= (*Z,Z*)-9,12-Octadecadienoic acid)	C18:2 D 9,12 cis	Omega-6	13.5	15.8
Stearic acid (=Octadecanoic acid)	C18:0D		0.5	0.2

### Cell viability and cytotoxicity

**Figure 1.**
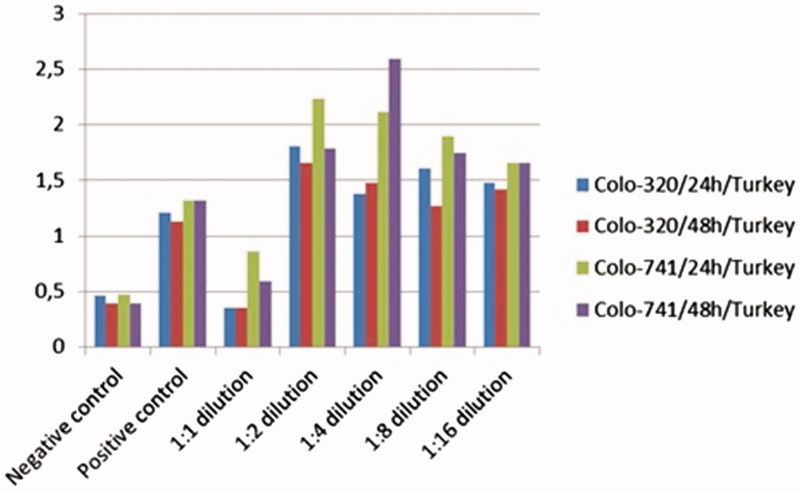
Effect of almond oil from Turkey on cell viability of Colo-741 and Colo-320 cells. Colo-741 and Colo-320 cells were treated with different concentrations of almond oil for 24 or 48 h. Viability was quantitated by the MTT assay.

Colo-320 and Colo-741 cells were treated with almond oil from NC and TR at various dilutions for 24 and 48 h. Cell viability was determined as described above by the MTT assay. Almond oil from TR inhibited the growth of Colo-320 and Colo-741 cells in a dose-and time-dependent manner. Our results showed that 1:1 dilution almond oil was more effective in inhibiting Colo-320 and Colo 741 cell growth when compared with other dilutions (Figure 1).

**Figure 2.**
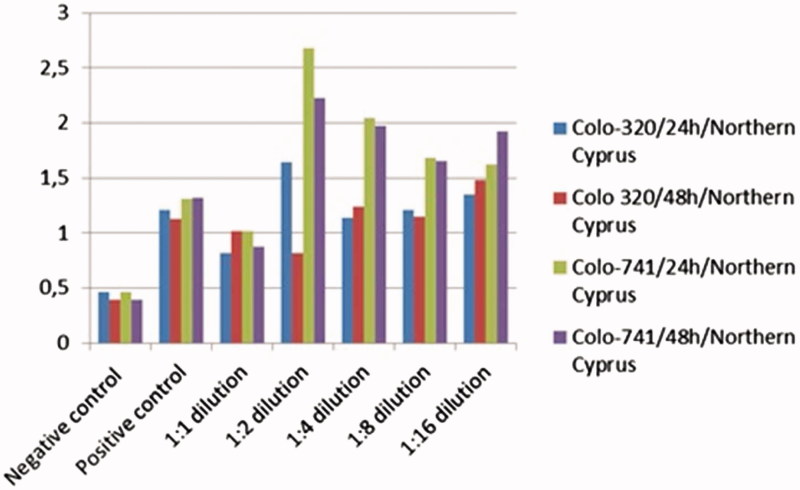
Effect of almond oil from Northern Cyprus on cell viability of Colo-741 and Colo-320 cells. Colo-741 and Colo-320 cells were treated with different concentrations of almond oil 24 or 48 h. Viability was quantitated by the MTT assay.

Additionally, growth and inhibition effects of almond oil NC to Colo-320 and Colo-741 cells (Figure 2) were observed in a dose- and time-dependent manner. Our results showed that 1:1 dilution almond oil was more effective in inhibiting Colo-320 and Colo 741 cell growth when compared with other dilutions. According to the MTT assay, results both almond oils NC and TR were used as a 1:1 dilution.

### Cell morphology

**Figure 3.**
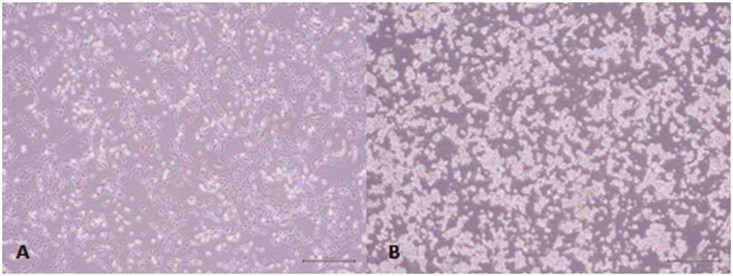
Colo-320 and Colo-741 cells imaged under an inverted microscope: (A) Colo-741 cells and (B) Colo-320 cells. Scale bars = 500 μm.

**Figure 4.**
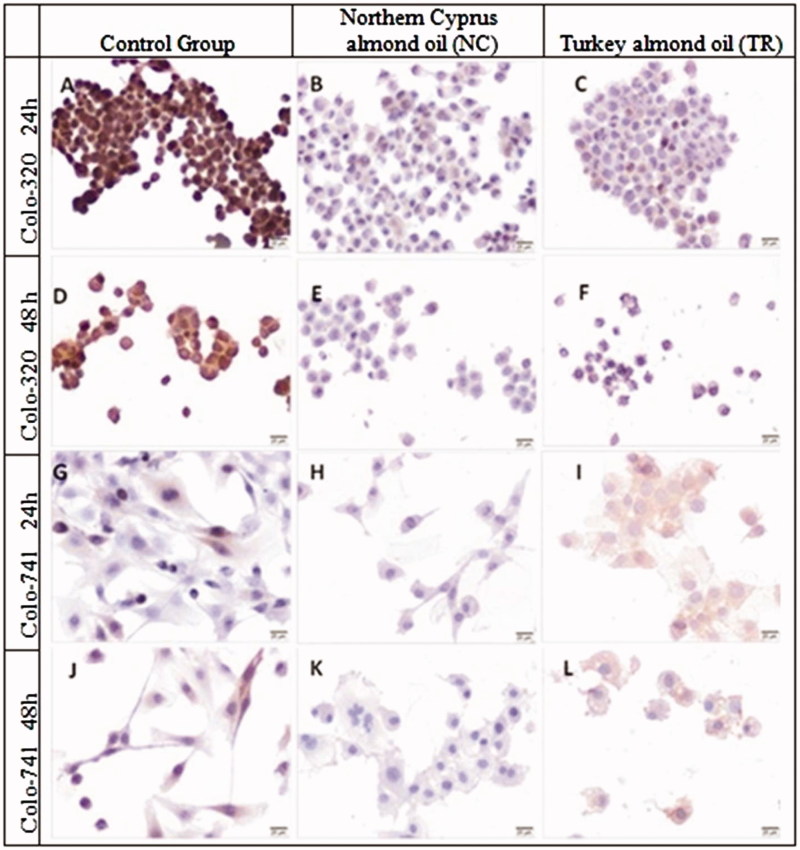
Immunoreactivity of β-catenin in Colo-320 (A–F) and Colo-741 cells (G–L) for 24 (A–C, G–I) or 48 h (D–F, J–L) culture with standard culture conditions (A, D, G, J) or Northern Cyprus (B, E, H, K) or Turkey (C, F, I, L) almond oils. Scale Bars = 20 μm.

Colo-320 cells are semi-adhesive, rounded and refractile cells in standard culture conditions (Figure 3(B)). After treating with both almond oils NC and TR, Colo-320 cells were shriveled and shrunken, especially when incubated with almond oil TR for 48 h (Figure 4(F)).

Colo-741 cells are fibroblast-like cells that grow with typical fibroblast colony morphology after 24 h in culture (Figure 3(A)). After treating with both almond oils NC and TR, the shapes of Colo-741 cells were changed to epitheloid-type cells after 24 h in culture (Figure 4(H,I)). When they were incubated 48 h, their shape was oval. In addition, vacuoles were detected in the cytolplasm of Colo-741 cells after both culture times with the two types of almond oils. This altered morphology was seen in Colo-741 cells treated with all almond oil. However, cells shriveled and shrunken were seen with Colo-741 cells treated with TR almond oil for 48 h (Figure 4(K,L)).

### Immunohistochemical evaluation

The H-SCORE of BMP-2, β-catenin, LGR-5, jagged 1 and Ki-67 immunolabelling in Colo-741 and Colo-320 cells treated with almond oils NC and TR for 24 and 48 h are given in Table 2. The immunoreactivity of β-catenin was significantly decreased in Colo-320 cells treated with almond oils NC and TR for 24 and 48 h compared to control group (Figure 4(A–C)); the intensity of β-catenin was significant in Colo-320 cells (*p* < 0.0001, Table 2). The immunoreactivity of β-catenin was higher in Colo-741 cells treated with TR almond oil for 24 h than NC almond oil (Figure 4(H,I)) (*p* < 0.0001, Table 2). The H-SCORE of β-catenin immunoreactivity was significantly decreased in Colo-741 cells which were treated with almond oil TR for 24, compared with 48 h incubated Colo-741 cells (Figure 4(I,L)) (*p* = 0.03, Table 2). The HSCORE of β-catenin significantly decreased in Colo-741 cells which were incubated with almond oil NC for 48 h but not for Colo-320 cells (Figure 4(E,F,K,L)).

**Table 2.** The H-Score of BMP-2, beta-catenin, LGR-5, jagged 1 and Ki-67 immunolabelling in Colo-741 and Colo-320 cells treated with Northern Cyprus and Turkey almond oils for 24 and 48 h.

		Control groups				Northern Cyprus almond oil				Turkey almond oil		
	Colo-320 cells		Colo-741 cells		Colo-320 cells		Colo-741 cells		Colo-320 cells		Colo-741 cells	
	24h	48h	24h	48h	24h	48h	24h	48h	24h	48h	24h	48h
β-catenin	440 ± 10	320.7 ± 15.89	217.7 ± 24.91	259 ± 12.12	207.7 ± 1.51	200 ± 0	202.3 ± 4.041	200 ± 0	222.7 ± 12.5	201.7 ± 2.887	295.7 ± 3,78	342 ± 36.37
BMP-2	465.7 ± 12.1	399.3 ± 1.155	266.7 ± 30.57	327 ± 11.53	316.3 ± 13.65	311.7 ± 4.726	312.3 ± 15.7	238 ± 11.14	209.3 ± 2.082	313.3 ± 5.686	363.7 ± 12,66	327.7 ± 47.92
Jagged 1	415.3 ± 14.57	449.7 ± 46.07	320.3 ± 13.28	316 ± 5.292	297 ± 41.76	304.7 ± 8.083	269 ± 14.53	217.7 ± 30.6	319.7 ± 23.71	298.7 ± 2.309	313.7 ± 8.08	256.7 ± 27.39
Ki-67	406 ± 3.464	375 ± 19.52	200 ± 0	200 ± 0	210 ± 0	404.7 ± 4.041	201.3 ± 2.309	200 ± 0	310,3 ± 3.512	207.3 ± 1.528	248 ± 15.59	200 ± 0
LGR-5	419.7 ± 2.517	429 ± 19.97	391 ± 7.81	348.7 ± 12.86	378.3 ± 61.16	404.7 ± 4.081	354 ± 8.544	367.7 ± 26.58	317.3 ± 20.21	334.7 ± 4.933	350.3 ± 13.8	221 ± 10.82
								58				

Data are expressed as means ± SD and were compared by ANOVA.

**Figure 5.**
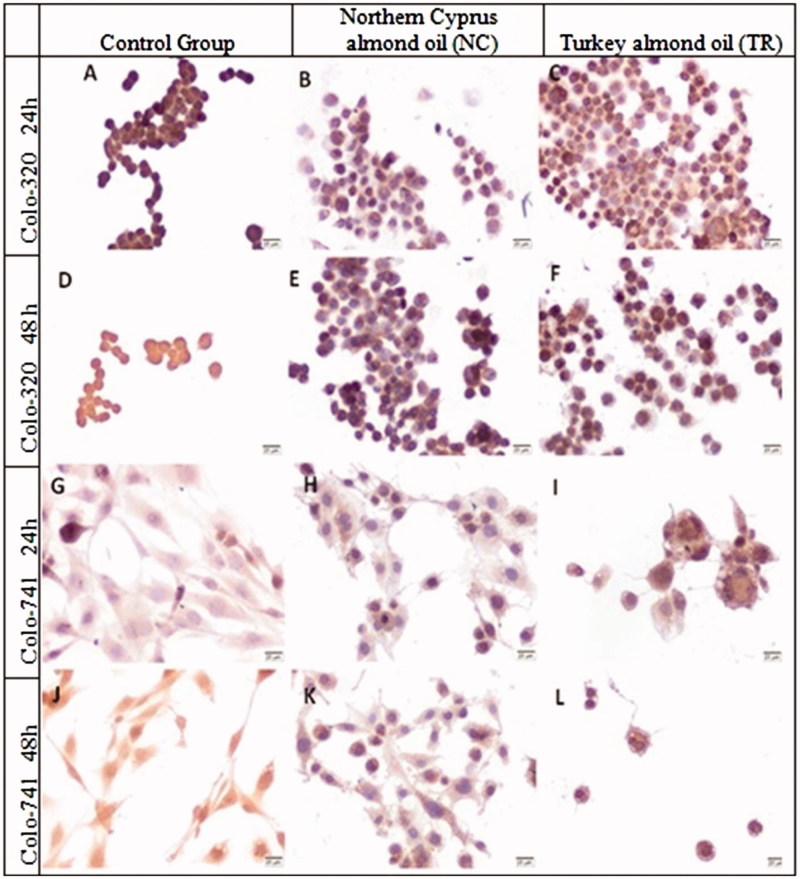
Immunoreactivity of BMP-2 in Colo-320 (A–F) and Colo-741 cells (G–L) for 24 (A–C, G–I) or 48 h (D–F, J–L) culture with standard culture conditions (A, D, G, J) or Northern Cyprus (B, E, H, K) or Turkey (C, F, I, L) almond oils. Scale Bars = 20 μm.

Immunoreactivity of BMP-2 was decreased in Colo-320 cells compared to the control group after treatment with both almond oils NC and TR for 24 h (Figure 5(B,C)) (*p* < 0.001, Table 2). The HSCORE of BMP-2 significantly decreased in Colo-320 cells which were incubated with almond oil TR for 24 h but this extract was not effective for BMP-2 expression in Colo-741 cells (Figure 5(B,C,H,I)). The BMP-2 immunoreactivity was similar in Colo-320 cells after incubation with almond oil NC for 24 and 48 h (*p* > 0.9, Table 2). However, almond oil TR treated Colo 320 cells showed higher expression of BMP-2 at 48 h compared with 24 h incubation (*p* < 0.001, Table 2). The BMP-2 H-SCORE of Colo-741 cells was significantly lower than Colo-320 cells after treatment with almond oil NC for 48 h (*p* = 0.0034, Table 2). The immunoreactivity of BMP-2 was higher in Colo-741 cells treated with almond oil TR for 48 h compared with those incubated with almond oil NC (*p* < 0.0003) (Figure 5(K,L)).

**Figure 6.**
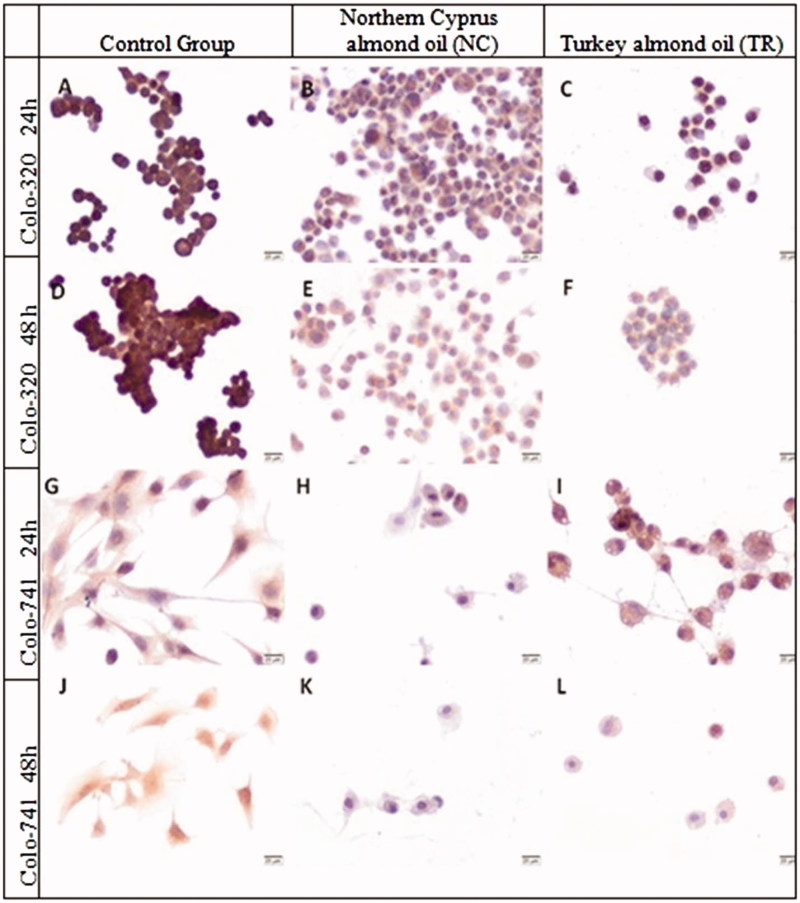
Immunoreactivity of Jagged 1 in Colo-320 (A–F) and Colo-741 cells (G–L) for 24 (A–C, G–I) or 48 h (D–F, J–L) culture with standard culture conditions (A, D, G, J) or Northern Cyprus (B, E, H, K) or Turkey (C, F, I, L) almond oils. Scale Bars = 20 μm.

As shown Figure 6, the immunoreactivities of jagged 1 in both Colo-320 and Colo-741 cells were less than control groups after treatment with pure almond oils NC and TR for 24 and 48 h (Figure 6). The immunoreactivity of jagged 1 was significantly decreased in both almond oils NC and TR treated Colo-320 cells compare to control groups but not for Colo-741 cells (Table 2). The jagged 1 immunoreactivity results did not differ in Colo-320 and Colo-741 after incubation with both almond oils TR and NC for 24 and 48 h (Figure 6, Table 2).

**Figure 7.**
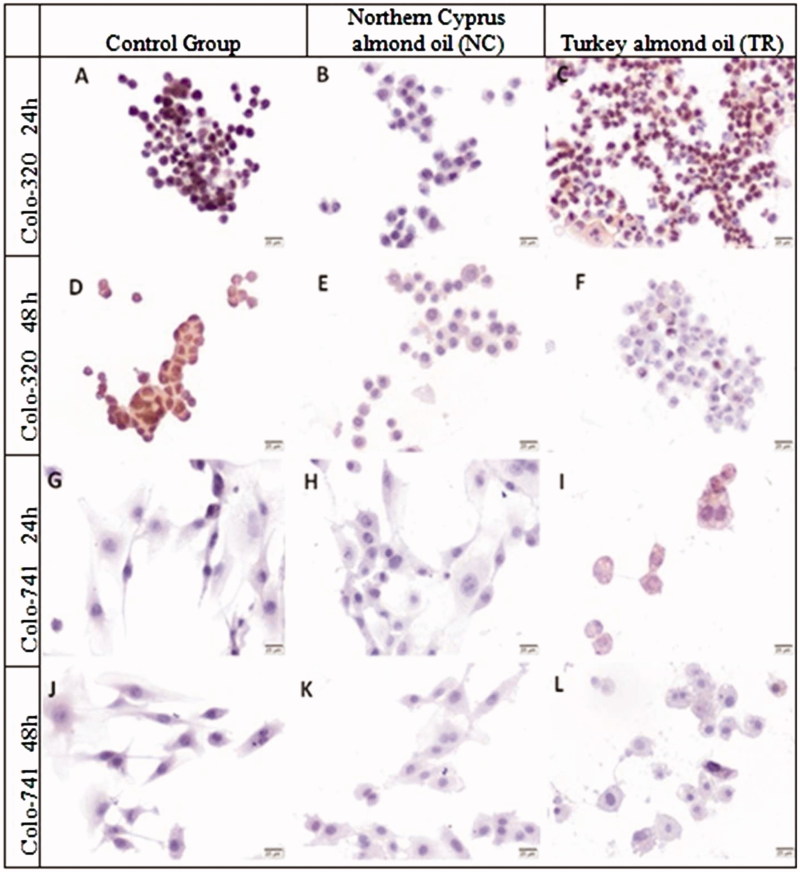
Immunoreactivity Ki-67 in Colo-320 (A–F) and Colo-741 cells (G–L) for 24 (A–C, G–I) or 48 h (D–F, J–L) culture with standard culture conditions (A,D,G,J) or Northern Cyprus (B, E, H, K) or Turkey (C, F, I, L) almond oils. Scale Bars = 20 μm.

Figure 7 shows that the H-SCORES of Ki-67 in Colo-320 cells treated with both almond oils NC and TR for 24 h were significantly decreased versus control groups (*p* < 0.0001, Table 2). The immunoreactivity of Ki-67 was significantly elevated in Colo-320 cells incubated with almond oil TR compare to almond oil NC (Figure 7(B,C)). The expression of Ki-67 was evaluable in Colo-320 cells and the median H-SCORE for 48 h was significantly higher than that of 24 h incubated cells (Figure 7(B,E)). Moreover, the Colo-320 cells treated with almond oil TR for 24 h showed a significant higher H-Score mean value in comparison with the Colo-741 cells (Figure 7(C,I)) (*p* < 0.001, Table 2). The immunoreactivity of Ki-67 was significantly decreased in both pure almond oil NC and TR treated Colo 741 cells compared to control groups (Figure 7(G,H,I)) (Table 2). The immunoreactivity of Ki-67 was higher in Colo-741cells treated with almond oil TR for 24 h than those incubated with almond oil NC (Figure 7(H,I)) (*p* < 0.0001, Table 2). In contrast to Colo-741 cells, Ki-67 immunoreactivity was significantly higher in Colo-320 cells treated with almond oil NC for 48 h compared with almond oil TR incubated cells (Figure 7(E,F)) (*p* < 0.001, Table 2).

**Figure 8.**
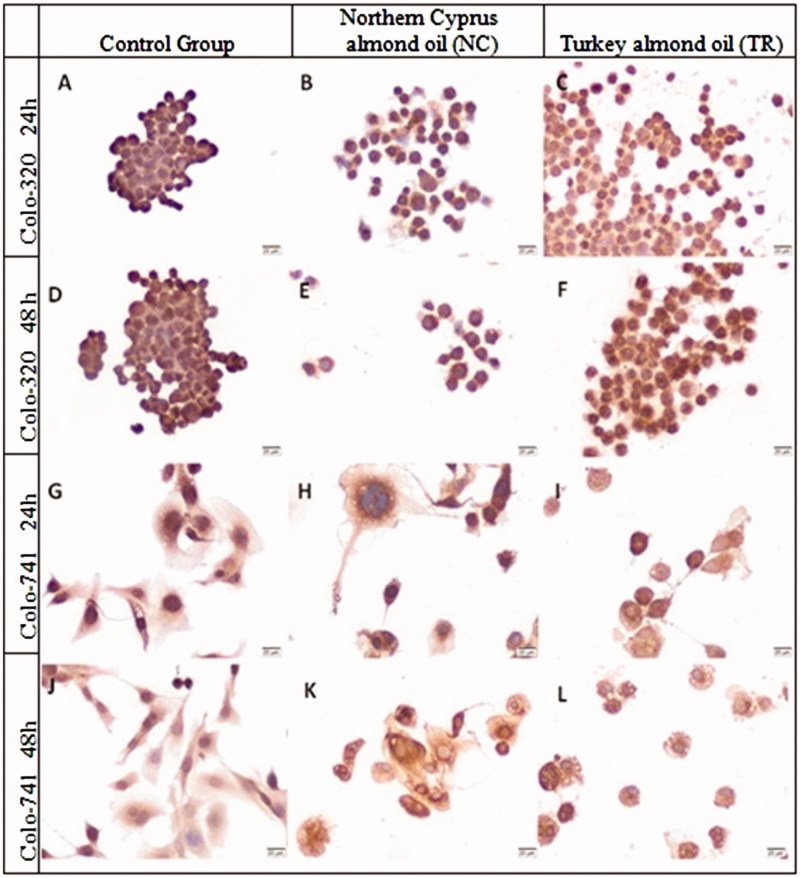
Immunoreactivity of LGR5 in Colo-320 (A–F) and Colo-741 cells (G–L) for 24 (A–C, G–I) or 48 h (D–F, J–L) culture with standard culture conditions (A, D, G, J) or Northern Cyprus (B, E, H, K) or Turkey (C, F, I, L) almond oils. Scale Bars = 20 μm.

Staining for LGR-5 in Colo320 and Colo-741 cells is summarized in Figure 8. The H-SCORE of LGR-5 significantly decreased in Colo-320 cells which were incubated with almond oil TR for 24 h (Figure 8(A,C)) (*p* < 0.0004, Table 2). The immunoreactivity of LGR-5 was significantly decreased in Colo-320 cells after incubation with almond oil TR compared to almond oil NC and control group (Figure 8(E,F)) (Table 2). LGR-5 immunoreactivity was significantly higher in Colo-741 cells treated with almond oil NC for 48 h compared with almond oil TR incubated cells, as like with Colo-320 cells (Figure 8(K,L)) (*p* < 0.0001, Table 2). The expression of LGR-5 was evaluable in Colo-741 cells treated with almond oil TR and the median H-SCORE for 48 h was significantly higher than 24 h incubated cells (Figure 7(I,L)).

## Discussion

The fixed oil yield of almond seeds and fatty acid composition of almond oil from Northern Cyprus were investigated for the first time in this study. According to the results of Askın et al. (2007) with 26 genotypes Turkish almond, seed oils were found to contain oleic acid ranging from 50.41 to 81.20%. Among the 26 samples, only 6 samples contained oleic acid more than an amount of 77%. This means almond seeds of Northern Cyprus are richer in oleic acid than 80% of Turkish almond samples.

Some studies showed that almond oil reduced colonic cancer incidence in rats (Davis & Iwahashi 2001). The specific effects of almond oil with respect to colon cancer cells remains undefined and there appears to be no published reports that have directly examined the effect of almond oil on colon cancer with *in vitro* and *in vivo* studies. In our study, the dilution of almond oil 1:1 was more effective than other concentrations in both types of colon adenocarcinoma cell lines. Therefore, the treatment dosage of the almond may be 1:1 when tested with *in vivo* studies. We also demonstrated for the first time that almond oil from Northern Cyprus (NC) and Turkey (TR) are an effective antiproliferative agent on both Colo-320 and Colo-741 cells. Therefore, the almond oils, both NC and TR, had similar effects in primary and metastatic colon carcinoma cells. Furthermore, we found that almond oil treatments had protective effects involving relevant molecular mechanisms, including BMP-2c, β-catenin, Jagged 1 and LGR-5, which are play a role during tumorogenesis.

The colon cells contain a gradient of molecular signal pathways including wingless-related integration site (Wnt), Hedgehog (HH), bone morphogenic protein (BMP) and Notch (Vinson et al. 2016). Notch and Wnt signalling are active in the base of undifferentiated colon crypt. However, BMP and HH signalling are active in the differentiated compartment of the crypt (Hardwick et al. 2008; Burgess et al. 2011). These signalling pathways play important roles in proliferation and differentiation of the colon crypt cells. In recent years, molecular signal pathways are increasingly attracting attention as a source of therapeutic targets for colon cancer (Qiao & Wong 2009). Primary and metastatic colon carcinoma may be controlled with different signalling pathways and transcription factors. In addition, these pathways or responsible molecules are still unknown. Also, using treatment procedures for both primary and metastatic colon carcinoma should be variable according to patient response and variation of cell types.

Jagged 1 is one of the ligands for Notch receptors and a pathogical link between Wnt and Notch pathways in colon cancer. Previous work showed that Notch activation, accomplished by β-catenin-mediated up-regulation of Jagged 1, is required for tumoroginesis in the intestine (Rodilla et al. 2009). In the current study, the immunoreactivities of Jagged 1 and β-catenin in both Colo-320 and Colo-741 cells were less than control groups after treated with pure almond oil NC and TR for 24 and 48 h. Additionally, almond oil NC exhibited a stronger inhibition effect on Jagged 1 and β-catenin than almond oil TR. This difference in the inhibitory effect is due to the variation in unsaturated fatty acid content of extracts, while the almond oil NC contains a high percentage of oleic acid which is a known compound with an anticancer effect in colon carcinoma cells (Carrillo et al. 2012; Burlamaqui et al. 2012; Davis et al. 2016).

The bone morphogenetic proteins (BMPs) play fundamental roles in embryonic development and control differentiation of a diverse set of cell types. The main role of BMP signalling in colorectal cancer neoplasia is poorly defined. A number of studies showed that abrogation of BMP signalling occurs during the progression of intestinal precursor lesions to cancers (Kodach et al. 2008a, 2008b). *In vitro* studies have been unable to clarify the functional significance of the BMP signal pathway in colorectal cancer. BMP-2 signalling inhibits self-renewal and induces differentiation of intestinal stem cells in colorectal cancer (He et al. 2004; Kodach et al. 2011; Lombardo et al. 2011). On the other hand, BMP signalling contributes to the progression of colorectal cancer by promoting invasiveness, epithelial–mesenchymal transition and tumour volume, following xenograft transplantation (Deng et al. 2007; Kang et al. 2009; Lorente-Trigos et al. 2010; Kim et al. 2015). Considering our results, which showed that BMP-2 protein immunoreactivity was different in Colo-320 cells, primer colon carcinoma cancer cells and Colo-741 cells, metastatic colon carcinoma cancer cells after treatment with both almond oils NC and TR. The immunoreactivity of BMP-2 decreased in Colo-320 cells which were incubated with almond oils TR and NC but increased in Colo-741 cells. It can therefore be concluded that BMP-2 expression and effects as either growth promoters or suppressors are different in diverse types of primary and metastatic colon carcinoma cancer cells. Thus, further *in vitro* work that utilizes colorectal cancer cell lines which may have a disrupted BMP signalling pathway as well as other mutations of key signalling components commonly found in colorectal cancer is required.

The leucine-rich repeat G protein-coupled receptor 5 (LGR-5), a target of Wnt signalling, is a marker of adult intestinal stem cells. Limited expression of LGR-5 was found in the crypt base of small and large intestines and was identified as the stem cell marker for cells capable of committed differentiation (Barker et al. 2007; Barker & Clevers 2007). Many studies have demonstrated that LGR-5 protein has a close association with initiation and repetition of different cancer types (Aoki et al. 2005; Tanese et al. 2008). LGR-5 protein was over-expressed in colorectal cancer and crypt LGR5 + stem cells in intestine have been observed to be represented as the cell of origin for intestinal cancer (Barker et al. 2009). Our study found that the immunoreactivity of LGR-5 was significantly decreased in both Colo-320 and Colo-741 cells incubated with almond oil TR compared to almond oil NC and control group. These data suggest that almond oil from both TR and NC may inhibit colon carcinoma cancer cell proliferation and tumour progression through obstructed expression of colon cell proliferation proteins.

To study cellular proliferation, immunoreactivity of the nuclear protein marker Ki-67 was used. This protein is a nuclear and nucleolar protein, which is tightly associated with somatic cell proliferation (Campelo et al. 2015). Our results show that the almond oil NC and TR have an antiproliferative effect on Colo-320 cells. The oil obtained from *Prunus dulcis* seeds was unable to affect the viability of Colo-741 cells while it exhibited cell morphology alteration.

## Conclusion

In conclusion, the fatty acid composition of almond oil of *Prunus dulcis* seeds collected from the western part of Northern Cyprus was investigated for the first time and unsaturated fatty acids (oleic acid percentage) was determined as higher than many others reported in the literature. Almond oils from Northern Cyprus (NC) and Turkey (TR) were investigated in both primary and metastatic colon carcinoma cells using different dilutions. Especially the 1:1 dilution was effective at both culture times and therefore may be used for *in vivo* studies. Almond oil containing unsaturated fatty acids, mainly oleic acid, have antiproliferative effects which decrease Ki-67 expression. Also, it decreased other signalling molecules which play a role during tumour viability metastasis and transcription factors. Especially, BMP-2 and β-catenin expression were affected after treatment of both type of cells.

Our results indicate that almond oils rich in oleic acid mediate anticancer effect with colon carcinoma cells. These *in vitro* results also need to be evaluated with *in vivo* experimental colon carcinoma models for further progress of almond oil, such as drug development, cancer prevention and usage as a complementary agent.
